# Infection with high proportion of multidrug-resistant bacteria in conflict-related injuries is associated with poor outcomes and excess resource consumption: a cohort study of Syrian patients treated in Jordan

**DOI:** 10.1186/s12879-018-3149-y

**Published:** 2018-05-22

**Authors:** Andreas Älgå, Sidney Wong, Muhammad Shoaib, Kalle Lundgren, Christian G. Giske, Johan von Schreeb, Jonas Malmstedt

**Affiliations:** 1 Department of Clinical Science and Education, Södersjukhuset, Karolinska Institutet, Stockholm, Sweden; 2 Médecins Sans Frontières, Operational Centre Amsterdam, Amsterdam, The Netherlands; 3 Médecins Sans Frontières, Amman, Jordan; 4 Department of Molecular Medicine and Surgery, Karolinska Institutet and Karolinska University Hospital, Stockholm, Sweden; 5 Division of Clinical Microbiology, Department of Laboratory Medicine, Karolinska Institutet and Karolinska University Hospital, Stockholm, Sweden; 6 Department of Public Health Sciences, Karolinska Institutet, Stockholm, Sweden

**Keywords:** Refugee, War wounds, Wound infection, Multidrug-resistance, Resource limited setting

## Abstract

**Background:**

Armed conflicts are a major contributor to injury and death globally. Conflict-related injuries are associated with a high risk of wound infection, but it is unknown to what extent infection directly relates to sustainment of life and restoration of function. The aim of this study was to investigate the outcome and resource consumption among civilians receiving acute surgical treatment due to conflict-related injuries. Patients with and without wound infections were compared.

**Methods:**

We performed a cohort study using routinely collected data from 457 consecutive Syrian civilians that received surgical treatment for acute conflict-related injuries during 2014–2016 at a Jordanian hospital supported by Médecins Sans Frontières. We defined wound infection as clinical signs of infection verified by a positive culture. We used logistic regression models to evaluate infection-related differences in outcome and resource consumption.

**Results:**

Wound infection was verified in 49/457 (11%) patients. Multidrug-resistance (MDR) was detected in 36/49 (73%) of patients with infection. Among patients with infection, 11/49 (22%) were amputated, compared to 37/408 (9%) without infection, crude relative risk = 2.62 (95% confidence interval 1.42–4.81). Infected patients needed 12 surgeries on average, compared to five in non-infected patients (*p* < .00001). Mean length of stay was 77 days for patients with infection, and 35 days for patients without infection (*p* = .000001).

**Conclusions:**

Among Syrian civilians, infected conflict-related wounds had a high prevalence of MDR bacteria. Wound infection was associated with poor outcomes and high resource consumption. These results could guide the development of antibiotic protocols and adaptations of surgical management to improve care for wound infections in conflict-related injuries.

**Trial registration:**

ClinicalTrials.gov (NCT02744144). Registered April 13, 2016. Retrospectively registered.

## Background

Worldwide, armed conflicts are a major contributor to the global burden of disease [1]. Conflict-related injuries often result in soft tissue and bone being contaminated with foreign material, leading to infection [2, 3]. The cornerstones of war wound management are surgical intervention and antibiotic therapy. Surgical interventions, mainly debridement of devitalized or infected tissue, are performed according to treatment principles for traumatic wounds [4]. Antibiotic therapy is utilised both as perioperative prophylaxis and as part of the treatment of wound infections [4, 5]. However, the increasing rate of antibiotic resistance globally [6], primarily owing to the over- and misuse of antibiotics [5, 7] threatens the effectiveness of available antibiotics. Retrospective reports of injured military combatants are the main source for knowledge about treatment efficacy in traumatic wounds [8, 9]. These results may not be applicable to a civilian setting due to the military use of ballistic protection and forward surgical teams. It is therefore unknown if wound infection itself threatens life and restoration of function for injured civilians.

Furthermore, it is unknown to what extent acute conflict-related wound infections are caused by multidrug-resistant (MDR) organisms. High incidence of MDR organisms in civilians has been shown in the Middle East, but reports generally are either based on culture results from chronic wounds [10, 11] or on all available culture samples, not limited to patients with clinical signs of infection [12, 13]. We had the unique opportunity to study cultures strictly obtained in the presence of clinical signs of wound infection in civilians with acute conflict-related injuries. The research on this patient group is limited. We aimed to assess MDR incidence and compare the outcome and resource consumption for acute conflict-related injuries, with and without wound infection in civilian patients receiving surgery. We hypothesised that infections negatively affect patient outcomes and lead to an increased burden of care.

## Methods

### Study setting

The Syrian armed conflict broke out in 2011 and quickly deteriorated, claiming 40% of all global war fatalities by 2012 [14]. Médecins Sans Frontières/Doctors Without Borders (MSF) runs an emergency trauma project at the Ministry of Health hospital in Ar Ramtha, Jordan, five kilometres from the Syrian border [15]. At this facility patients from the Syrian armed conflict receive treatment for blast and gunshot injuries. Wounds are treated according to guidelines based on the International Committee of the Red Cross (ICRC) war surgery protocol [4]. As adjunct to surgical wound debridement, all patients receive prophylactic treatment for 48–72 h with narrow-spectrum antibiotic agents (Cefazolin or Ampicillin). Metronidazole is added for patients with complicated open fractures, perforated viscous at laparotomy or penetrating craniocerebral wounds. Acute war wounds are left open following the first surgery. After 3–5 days, wounds are assessed and treated by delayed primary closure if possible.

### Study design and participants

We performed an open cohort study with longitudinal data collection. We used routinely collected clinical data from consecutive patients admitted due to acute conflict-related injuries. Availability of standardized culture results determined the start of the study (September 17, 2014), and closure of the Syrian border determined the end (June 21, 2016). The cohort included all patients that received surgical treatment for conflict-related wounds. Surgeons obtained intraoperative tissue samples (bone, soft tissue, or fluid) only if the wounds showed clinical signs of infection. An accredited clinical microbiology laboratory (King Abdullah University Hospital, Ar Ramtha) performed culture and antibiotic susceptibility testing. Main culture media were blood, chocolate, and MacConkey agar. Species were identified through manual flowcharts or automated Vitek^®^ 2 technology (bioMérieux, Marcy l’Etoile, France). Antibiotic resistance was determined by manual disk diffusion (Bio-Rad, Hercules, California, USA) or automated using Vitek^®^ 2 technology, and interpreted according to the guidelines from the Clinical and Laboratory Standards Institute [16]. We defined clinical signs of infection as purulent discharge [17, 18] or foul smell, in accordance to locally established clinical routines. The diagnosis of wound infection required both clinical signs of infection and at least one positive culture.

Paper-based patient files provided information on vital signs at hospital admission. We calculated coded Revised Trauma Score (RTSc) [19] from respiratory rate, systolic blood pressure, and Glasgow Coma Scale [20] in a subsample consisting of all patients with infection (cases) with one non-infected control matched for age and sex. Mean RTSc in cases was then compared to controls to investigate possible confounding by patients with infection having sustained a more severe trauma compared to those without.

The clinical patient database provided demographic information, admission dates, diagnoses and details of performed surgeries. Wound culture reports provided details of bacterial species and their resistance patterns. We defined MDR as resistance to at least one antibiotic from three or more relevant antibiotic groups [21]. We considered methicillin-resistant *Staphylococcus aureus* (MRSA) as MDR.

The distinction between combatants and civilians is complex, particularly in internal armed conflicts [22]. Since our aim was to study non-military injuries, we defined civilians as patients without protective equipment, such as body armour or helmets, and without assistance by forward surgical teams.

### Outcomes

The outcome measures were in-hospital death, limb amputations and resource consumption (number of surgeries, length of hospitalization).

### Statistical analysis

We used the Epidata entry software (The Epidata Association, Odense, Denmark) to collect paper-based clinical, and microbiology data, and performed all analyses using SPSS Statistics (IBM Corp., Armonk, NY, USA). We used mean with standard deviation (SD) for summarizing scale variables, and proportions with 95% confidence intervals (95% CI) for categorical variables. We conducted bivariate analyses with Chi-square to compare two categorical variables, and t-test to compare scale variables. We used binary logistic regression models to evaluate differences in outcome and resource consumption between patients with and without infections. We considered *p*-values < .05 significant.

### Role of the funding source

The funders of the study had no role in study design, data collection, data analysis, data interpretation, or writing of the report. Authors had access to the raw data in the study, and had final responsibility for the decision to submit for publication.

## Results

The cohort consisted of 457, predominantly male (86%) individuals with a mean age of 27 years (95% CI 25–28) (Table 1). Clinical signs of infection were found in 81/457 (18%) patients. Wound infection was verified with at least one positive culture in 49/81 (60%) patients, corresponding to a wound infection rate of 11% (49/457). During the study period 37/457 (8%) patients died in hospital and 48/457 (11%) patients had a major amputation.

**Table 1:** Characteristics of 457 consecutive patients that received surgical treatment due to conflict-related injuries

Characteristic		
Age in years at hospital admission, mean (95% CI)	27	(25–28)
Male, n (%)	395	(86)
Discharged, n (%)	198	(43)
Defaulter, n (%)	23	(5)
Referred to other structure, n (%)	199	(44)
Death, n (%)	37	(8)
Number of readmissions, n (%)		
0	400	(88)
1	45	(10)
2	10	(2)
3	2	(0)

MDR was detected in 36/49 (73%) of patients with positive wound cultures. Mean time to detection of MDR bacteria was 16.5 (IQR 8–30) days after hospital admission. The most common bacteria were *Staphylococcus aureus* (15/49) (73% MRSA)*, Pseudomonas* (12/49) (17% MDR), *Klebsiella pneumoniae* (11/49) (82% MDR), *Enterobacter* (9/49) (78% MDR), *E. coli* (8/49) (100% MDR), *Proteus* (8/49) (63% MDR), and *Acinetobacter* (5/49) (100% MDR)*.* The *K. pneumoniae*, *Enterobacter*, and *E. coli* strains were resistant to most antimicrobials, but remained susceptible to carbapenems. Most of the *Acinetobacter* strains were also resistant to carbapenems.

Mean RTSc was similar in matched patients with (7.31, SD 0.67) and without (7.33, SD 1.18) infection, indicating that patients with infection were not more severely injured than patients without infection. The most common surgical procedure was debridement of tissue (39%), followed by laparotomy (11%), and external fixation of fracture (10%) (Table 2).

**Table 2:** Number and type of primary procedure

Type of primary procedure	n (%)
Debridement of tissue	180 (39)
Laparotomy	49 (11)
External fixation of fracture	47 (10)
Wound closure	33 (7)
Vascular repair	31 (7)
Amputation	25 (5)
Other orthopaedic procedure	21 (5)
Chest procedure	19 (4)
Other surgical intervention	17 (4)
Bowel repair	16 (4)
Skin graft	12 (3)
K-wire manipulation	4 (1)
Solid organ management	3 (1)
Total	457 (100)

### Death

Of patients with infection 2/49 (4%) died, compared to 35/408 (9%) of patients without infection, crude relative risk = 0.48 (95% CI 0.12–1.92) (Table 3). The two patients with infection that died did so after 40 and 49 days; whereas patients without infection died after a median time of 5 (IQR 2–14) days. Of patients with MDR infection 2/36 (6%) died, compared to 0/13 (0%) of patients with non-MDR infection.

**Table 3:** Outcomes in patients with and without wound infection

Outcome	With infection (*n* = 49)		Without infection (*n* = 408)		p
Amputation, n (%)	11	(22)	37	(9)	.007^a^
Death, n (%)	2	(4)	35	(9)	.407^a^
Amputation or death, n (%)	11	(22)	66	(16)	.311^a^
Days in hospital, mean (SD)	77	(50)	35	(39)	< .0001^b^
Number of surgical procedures, mean (SD)	12	(9)	5	(5)	< .0001^b^
Major surgical procedures, mean (SD)	11	(7)	4	(4)	< .0001^b^

*SD* standard deviation

^a^ Chi^2^-test

^b^ t-test

### Amputation

Patients with infection had a higher amputation rate (22%), compared to patients without infection (9%) (Table 3), crude relative risk 2.62 (95% CI 1.42–4.81). Patients with MDR infection had a slightly higher risk for amputation than patients with non-MDR infection, although non-significant (9/36 (25%) compared to 2/13 (15%), relative risk 1.83 (95% CI 0.34–9.89)).

### Resource consumption

The average numbers of procedures per patient were six (95% CI 1–47). Patients with infection needed 12 surgeries on average, compared to five in patients without infection (Table 3). Patients with MDR infection needed 13 surgeries on average, compared to 11 in patients with non-MDR infection (*p* = .690).

Mean length of stay was longer for patients with infection (77 days), compared to patients without infection (35 days), (*p* < .000001). Mean length of stay was 69 days for patients with MDR infection, and 99 days for patients with non-MDR infection (*p* = .131).

### Multivariate analysis

The increased risk for amputation in patients with infection remained after adjustment for sex and age, odds ratio 3.0, 95% CI 1.40–6.43 (*p* = .005).

## Discussion

Wound infection was associated with poor outcomes and high resource consumption in this cohort of Syrian civilians who had surgery for acute conflict-related injuries. We reduced the risk for misclassification by using appropriate criteria for wound infection, i.e. clinical signs of infection verified by a least one positive tissue culture. To our knowledge, this is the first large cohort study investigating the association between wound infection *and* outcome and resource consumption in civilians with conflict-related injuries.

Patients with infection needed more surgeries, including amputations, compared to those without. Previous research on military combatants with open tibia fractures have found infection to be significantly associated with both amputation and need for surgery [23], while another study on military combatants was not able to show this association [24]. The average number of surgeries in the present study was six (12 for infected wounds), which highlights the complexity of care. The ICRC war surgical manual suggests that more than two surgeries denote a complication [4]. Our study results contradict this low “benchmark” number. Moreover, mean length of stay was longer for patients with infection compared to patients without infection, which is in concordance with previous results from both military [23] and civilian settings [25].

We found an unexpectedly high occurrence of MDR organisms in acutely injured civilians with wound infection (73%). Since MDR infection tends to develop over time, we expected lower MDR levels than those of previous reports of Syrian civilians with non-acute conflict-related injuries in Jordan (69%) and Israel (59%) [11, 12]. A high prevalence of MDR bacterial colonization has also been shown when screening conflict-injured civilians in Libya [26], as well as after having fled to Europe (59%) [27]. MDR bacteria, particularly those forming biofilm, has been identified as a significant risk factor for persistent infection in patients with conflict-related wounds [28]. Since patients in our study were not screened for MDR bacteria it is difficult to determine whether they were colonized at admission or acquired MDR bacteria during hospitalization. Our results suggest an association between MDR infection and amputation, however non-significant. This lack of significance could be due to the high incidence of MDR infection. This is supported by results from a study with a lower MDR incidence (32%), where only 2% were amputated [29].

The high prevalence of MDR in Syrian civilians may be partly explained by a wide availability of over-the-counter antibiotics in Syria prior to the armed conflict [30]. Colonization of the patients themselves and healthcare-associated infections have been suggested as the main source for conflict-related wound infections by MDR organisms [31]. Simultaneously, it is crucial to distinguish infection from colonization to limit the use of broad-spectrum antibiotics, since excessive use may lead to further development of MDR organisms [32].

Knowledge on the likelihood of, or presence of, infection in wounds treated in conflict-related settings may potentially lead to alterations of protocol. According to the commonly used ICRC regimen, wounds are debrided surgically and a narrow spectrum antibiotic agent is supplied at the time of debridement. Our results show that presence of infection is associated with prolonged hospitalization, indicating that debridement was not curative in most infected patients. The high prevalence of MDR bacteria suggests that routine narrow spectrum prophylaxis may be insufficient in settings similar to that at Ar Ramtha Hospital, Jordan. Additionally, routine use of narrow spectrum antibiotics could contribute to an increase of MDR, which suggests a need for caution in a setting where three of four patients with wound infections are infected by MDR bacteria.

Our results should be interpreted taking into consideration some limitations. First, the dependence upon routine data limited the number of parameters available for control of confounding. We lacked details on comorbidities relevant for wound healing. However, with the cohort mean age of 27, chronic diseases, such as type 2 diabetes, are likely to be rare. Second, it is difficult to exclude that some cultures were collected from patients with limited clinical signs of infection. Local treatment guidelines state that cultures should only be obtained from wounds with clinical signs of infection. We performed semi-structured interviews with the treating physicians and found satisfactory adherence to these guidelines (data not yet published). Third, we risk underestimating the frequencies of wound infection with our strict definition, requiring both clinical signs and at least one positive culture. However, the risk of bias is thought to be low, as under-diagnosis of infection probably is non-differential and therefore not related to outcome. The major limitation is the lack of detailed information on injury severity. Although we calculated RTSc scores for each clinically infected patient and for matched non-infected patients and found no significant difference between the two groups, we lack anatomical and wound-specific details, such as localisation and extent of injury. We cannot exclude that such parameters differ between infected and non-infected patients. Strengths of the study include the prospective approach, a large cohort of consecutive civilian patients with acute conflict-related injuries, the clear distinction that cultures originate from patients with a clinically detected wound infection, intraoperative tissue sampling for culture and the use of an accredited microbiology laboratory.

## Conclusions

Wound infection is associated with poor outcomes and high resource consumption in Syrian civilians with conflict-related injuries. Patients with infection needed more surgeries, underwent more amputations, and were hospitalized longer, compared to those without infection. In addition, we found a high occurrence of MDR organisms in patients with infected conflict-related wounds. Our results could increase the understanding of context-specific MDR patterns, and aid the development of antibiotic and surgical protocols. These actions could potentially help clinicians to reduce serious wound infections and decrease morbidity. Future research is needed to prove that the association between wound infection and outcome represents causality. We urge for more studies to define what is an acceptable re-operation rate in a conflict setting.

## Abbreviations

CI: Confidence interval
ICRC: International Committee of the Red Cross
MDR: Multidrug-resistance
MRSA: Methicillin-resistant *Staphylococcus aureus*
MSF: Médecins Sans Frontières/Doctors Without Borders
RTSc: Revised Trauma Score, coded
SD: Standard deviation
